# 6 Feet Apart: Spaces and Cultures of Quarantine

**DOI:** 10.1177/1206331220938622

**Published:** 2020-08

**Authors:** Rob Shields, Michael Schillmeier, Justine Lloyd, Joost Van Loon

**Affiliations:** 1 University of Alberta, Edmonton, AB, Canada; 2 University of Exeter, Exeter, UK; 3 Macquarie University, North Ryde, New South Wales, Australia; 4 Katholische Universität Eichstätt-Ingolstadt, Eichstaett, Freistaat Bayern, Germany

**Keywords:** COVID-19, pandemic, space-time, spatialization, globalization

## Abstract

Introduction to Spaces and Cultures of Quarantine. This special issue assembles a set of short interventions selected by internal blind review from submissions in response to a call for papers. The contributors document the first phase of the pandemic from February to May 2020, reflect on and respond to the first few months of the global spread of COVID-19, its arrival in communities and its personal impacts and effects on the public realm, from travel to retail to work and civil society. They encompass many continents, from Latin America to Asia. Staying six feet apart provides a rubric for the spatial experience and impact of the COVID-19 pandemic on urban life, our understanding of public interaction, crowd practice, and everyday life at home under self-isolation and lockdown. Time changed to a before and after of COVID-19. The temporality of pandemics is noted in its present and historical popular forms such as nursery rhymes (Ring around the Rosie). Place ballets of avoidance, passing by, long days under lockdown and hurried forays into public places and shops create a new social performativity and cultural topology of care at a distance.

## Introduction and Call

Staying six feet apart to avoid infection is just the tip of the lived experience of the pandemic, quarantine, and isolation. Enforced and voluntary forms of geographical quarantines, new protections such as facemasks, and mobile avoidances produce new public behaviors and crowd practices. “Six feet apart” reshapes or “refigures” not only public and private interaction but also generates online and face-to-face publics (Dewey, 1954) that must engage with the risks, uncertainties, and requirements of the COVID-19 situation. COVID-19 changes the human condition. It has transformed time-space “cultural topologies” of social and environmental relations at all scales, locally and globally (see Shields, 2013). Every child, parent, and elderly person on the planet is affected by the pandemic. Even if not (yet) infected, they have been palpably affected by this social refiguration with an immediacy and speed that is unprecedented in human and possibly in geological history.

This special issue of *Space and Culture* assembles a set of short interventions. These were selected by internal blind review from submissions in response to a call for papers that closed May 15 2020. Thanks in part to DeMond Miller recalling a previous rapid response issue in the aftermath of Hurricane Katrina (*Space and Culture* 9:1, 2006). The contributors reflect on and respond to the first few months of the global spread of COVID-19, its arrival in communities, and its personal impacts and effects on the public realm, from travel to retail to work and civil society. They encompass many continents, from Latin America to Asia. This Introduction cites the contributions that follow in this issue by author surname only without publication date. As documents of the first phase of the COVID-19 pandemic (January–May 2020), they reflect the partialness of the understanding of the virus and pandemic as well as ambiguities about the local rates of transmission intersected by an unprecedented amount of media panic and repetitive general information. At this time, public discourse seemed limited by a lack of investigative access and the colonization of disputes about the virus by politicians and media pundits who sought to enhance their public visibility with daily press conferences that were often uninformative distractions. Indeed, keeping up with daily media coverage became an obsessive source of anxiety for many people cooped up under new norms of self-isolation and local social policies of lockdowns. Neoconservative ‘Strong-men’ leaders defied advice and sacrificed the vulnerable. In sharp contrast, in New Zealand, East Asia, and most of continental Europe, virologists, immunologists, and epidemiologists dominated the public sphere, as politicians dared not defy science-based insight.

## The Ethics of Quarantine Spaces and Mobilities

Quarantine is a symptom of deeper problems in globalized socio-economic relations, which Maffesoli argues are profoundly civilizational. Van Assche affirms that we need new approaches to socio-ecological governance. We share our space with a virus, which binds us into the sociality of the SARS-CoV-2 positive and slays many of us. Although some, such as the elderly, the disabled or the immunocompromised are more at risk than others, such as youth, predicting the path of contagion has been difficult and the severity of its symptoms are capricious, which results in the appearance of complexity and the rise of conspiracy theories and almost random seeming treatments such as taking the anti-malaria medication hydroxychloroquine.

The COVID-19 world alludes to the fantastical, because it is beyond imagination, the stuff of unpredictable nightmares and phantasms such as Reis-Filho finds for zombie films as metaphors of contagion. Modern versions of the 14th century quarantines are explored by many contributors such as Tuncbilek. These present new faces of isolation and spatial segregation. Both Reis-Filo and Cano-Hila notes that zombie films have been a popular trope as collective being-together became a fearful being-against for some because there appeared to be no “safe space.” Quarantine forked to become both imposed by authorities on individuals whose exposure status was unknown, alongside a self-directed physical distancing that relied on masks, keeping some sort of distance from others apart from household members, and retrospective decontamination by soap washing hands for 20 seconds. Staying home, wearing masks, and washing hands to protect vulnerable others were global performances connected with a largely voluntary quarantine but also forced confinement in some jurisdictions (Gromova 2019; Twilley and Manaugh 2014).

At the same time as segregation, quarantine in the form of staying-at-home brought neighbors and members of households and families into more frequent contact. Although separated by walls or fences, the soundscape of apartments, back gardens, and city streets changed (see Flynn, 2020). Less traffic allowed not only natural birdsong to be heard better but also neighbors’ laughter, conversations and nearby everyday activities. The resonant frequencies of cities changed as transportation was reduced, buses idled, aircraft grounded due to the risks associated with travel and fears of public and any sort of shared transport (Viderman). Everyday life includes not only solidarity but conflicts and resentful adjustments to noise, especially for activities that do not align in time such as a neighbor who does construction work during one’s own mealtime.

In public, stores, and other semi-public locations, scarves and masks have been adopted, even in jurisdictions that had banned face coverings as a result of anti-Islamic antagonisms (see Stansbury 2020). Masks have often been homemade due to lack of supply. These “do it yourself” adaptations of underwear, vacuum cleaner bags, and hand-sewn fabric masks represent a new material vernacular of COVID-19. Vallee notes how they changed our voices. The performativity of proxemics made hygiene suddenly become of intense shared concern. This also produced new moral panics and ethical divisions: Those wearing masks versus those not (and similarly those wearing gloves and those not); those standing too close or stopping to rest in parks; those following one-way arrows in store aisles versus those not.

Physical distancing by staying six feet apart became both a novel social discussion and a new social grammar. Mickey Vallee has commented that, like a flock of starlings, human murmurations were changed as each individual attempted to maintain a physical distance from those outside of their own households while out in public. However, the necessary distance were always a matter of individual interpretation and depended on performance “on the fly”. Avoidance has often required stepping off pathways and a new attention to proxemics, and revealed the shortcomings of streets organized around automobile priorities and too-narrow pavements and walkways that had been reduced to ergonomic minimums in the name of modern efficiency. The COVID-19 “place-ballet” (Buttimer & Seamon, 1980) of the streets required a new spatialization, new levels of attentiveness, and performativity to accomplish a care for others, as Russel and Vannini note. This is not mere gallantry, but politeness in a “risk society” (Beck, 1992), a new public manner that reduced spontaneity and certainly avoided “bumping into” others.

Barbosa and other contributors note that crisis is a familiar condition for many in the Global South and we risk not hearing their comments. Badilla Rajevic details how Chilean gatherings were refashioned and protests in public places demobilized. What Löw refers to as a “refiguration” of social space also has a novel corollary in the time dimension. Once workplaces reopened to those who must necessarily be present in person, phased or sequenced start times at work, it allowed the numbers taking elevators at any one time to be reduced. In public, people are exhorted to stay six feet apart and not to loiter in but walk through parks as a way of preventing congregations of friends. Getting together became a precious memory. The gravity of public gatherings was highlighted. Later in the pandemic, crowds defied public health advice globally to protest black oppression and racialized policing after the death of George Floyd in Minneapolis.

In a pandemic situation, bodies are suspect because they are involuntary toxic media of infection. Contact between bodies becomes a feared danger that actualizes the risk that is widely felt and discussed. Transmission of the virus across the boundaries of the body and through breath reminds us of the conceit of imagining our own purity and individual separateness and independence from the environment (Bogdan, 2020; Mbembe, 2020). As our call for papers noted, it also highlights a sociology of “the ways we are or are not already bound, and for whom those binds and boundaries are chains and chokeholds” (Sophie Lewis quoted in Lennard 2020) such as those forced to report to work for the convenience of others. Boano places the differential vulnerabilities and radical inequalities and violence of quarantine at the center of the urban question of quarantines. Guevara and Vargas contribute the perspective of the marginalized in Bogotá on infrastructures of care and resilience.

## Quarantine Time and Viral Tempo

There is a global before and after of the pandemic. COVID-19 has become a historic dividing line and also “time” itself, in the sense of an age or epoch. It is not only definitive of social and economic life but drags on with a frustrating persistence as Morrow and many other contributors note. Across the literature of COVID-19 and its commentaries, its temporality is felt or expressed in negative effects. So many have commented on the sensation of time having stopped or life under covid restrictions moving at a slower pace or different rhythm. For many, the hours of work continued with dismal regularity on videoconferencing and home computers. Iranmanesh critiques the substitution of digital virtuality for public space. The diurnal cycle of work and private time was suspended in favor of blended work from home or pure isolation for weeks. New daily benchmarks took the place of previous routines, such as family walks, mealtimes, or measuring progress toward a goal or a personal “lockdown project.” For some living alone, pets were more than ever the closest companions. Valizadeh argues that quarantine is not only a spatialization of isolation but that as time “stops” it includes a suspension of our shared social time.

COVID-19 acquired distinctive temporal notes lived through rituals such as the time of popular songs. Most famously amongst several alternates, “Happy Birthday” (sung twice through) found a new use as the anthem of 20 seconds of handwashing. Gloria Gaynor’s “I will survive” lip-synced in bathrooms and circulated in short videos proved a popular rival (Bruner, 2020). However, no ubiquitous lyric has yet emerged for the current pandemic. Instead, the Italians of Lombard towns, singing together from balconies that became the defining musical note (see Fiumi, 2020). People have turned to re-listening to older music, but no single song compares today with the traditional English nursery rhyme that dates back to the Black Death (1347–1351). One version is:

Ring around the Rosie
Pockets Full of Posies
Ashes, Ashes
We All Fall Down!

For many childhood memories, this rhyme was usually recited together sing-song style, dancing in a sort of “musical gesture” (Hatten 2004; Mazzola & Andreatta, 2007) of holding hands and circling around, then falling down to the mirth of all. This kinesthetic coordination and togetherness make morbid fun of collective death, drawing it into the circle of life and the vitality of laughter, and the rhythmic recitation of the rhyme and turning tropes of death into play.

Six feet apart sums up a precarious social dance, a place-ballet of avoidance, passing by and temporal rhythms of long days under lockdown, hurried forays into the public, and the quickened step aside to stand back from passersby. In this new social performativity and cultural topology, care at a distance on videoconferences, phone, and social media has had to substitute for our vigils beside the dying and attending to the frail, accepting embraces, and offering comfort to friends and loved ones.
